# Experimental Researches Applied to Physiology and Pathology

**Published:** 1856-05

**Authors:** E. Brown-Sèquard


1. *Experimental Researches applied to Physiology and Pathology.* By E. BROWN-SÈQUARD, M. D. Pp. 124. New York. 1853.
2. *Recherches Expérimentales sur la transmission croisée des impressions sensitives dans la moelle épinière.* Par le Docteur E. BROWN-SÈQUARD. Pp. 19. Paris. 1855.
3. *Propriétés et Fonctions de la Moelle Epiniére.* Rapport sur quelques Expérience de M. BROWN-SÈQUARD. Par M. PAUL BROCA. Pp. 66. Richmond (U. S.) 1855.
4. *Experimental and Clinical Researches on the Physiology and Pathology of the Spinal Cord, and some other parts of the Nervous Centres.* By E. BROWN-SÈQUARD. Pp. 66. Richmond, (U. S.) 1855.
5. *Des Phénomenes de Contraction Musculaire observés chez des individus qui ont succombé à la suite du choléra ou de la fièvre jaune.* Thèse, par le Dr. GEORGE HENRY BRANDT. Pp. 43. Paris. 1855.

Startling results have followed the vivisections performed by M. Brown-Séquard, in respect of the Physiology of the Spinal Cord; and it is to these, and to the support which his conclusions derive from pathological observations, that we would at present direct our readers' attention.

It has long been a matter of belief that, while the anterior columns of the spinal cord conducted voluntary motor influences downwards to the muscles on their own side of the body, the posterior columns and part of the lateral were concerned in carrying upwards sensorial nervous impressions also from their own side. From the experiments of M. Brown-Séquard dates a new era in the physiology of the spinal cord; and we will endeavor, as briefly as we can, to point out what he has proved by careful and repeated sections in various ways, of the spinal cord, in a variety of animals; sections made in the presence of numerous distinguished physiologists, who were on the alert to detect fallacy, and who have reported most favorably on the correctness of the conclusions drawn.

*Results of lateral section of the spinal cord.*—This section, made so as to divide the whole of one half of the cord (*i. e.*, one anterior, lateral and posterior column) shows that the *nervous fibres which conduct sensory impressions cross or decussate all along in the spinal cord.* Hence it happens that, when a section of this kind is made, sensibility is lost on the *opposite* side, below the section; while it is not lost, but in fact is exaggerated, on the same side as the section, which only exhibits the well-known paralysis of motion.

The impressions on the left side of the body then are transmitted (mostly) by the right side of the spinal cord, and *vice versa.*

When a longitudinal section is made along the median line of the cord, at the part giving nerves to the lower extremities, the decussating fibres of both sides are necessarily divided, and sensory paralysis affects both lower extremities, while power of voluntary motion remains. A very curious result has been further observed in the instance of animals which have undergone the lateral section, namely, that they have become subject to a convulsive affection resembling epilepsy, but differing from it in the evident absence of loss of consciousness.

Section of a lateral half of the *medulla oblongata* gives, as regards sensibility, the same results as section of a lateral half of the spinal cord.

With respect to the seat of the decussation of the *voluntary motor fibres,* M. Brown-Séquard holds the opinion that in man, while it cannot be maintained that they *all* decussate in the medulla oblongata, where the fibres of the lateral columns cross each other to form the anterior pyramids, yet that the number of these fibres which do not decussate there is certainly small.

In the fourth work on the list at the head of this article, the correspondence of these views with pathological observations is most clearly established. "According to the seat of an alteration in the cerebro-spinal axis, producing a paralysis, there are three different kinds of paralytic effects which may exist: *a.* The alteration being in any part of the encephalon, except the inferior part of the medulla oblongata,—the paralysis of voluntary movement and of sensibility, exists in the side of the body opposite to the side of the disease. *b.* The alteration occupying an entire lateral half of the inferior portion of the medulla oblongata, at the level of the decussation of the pyramids,—the paralysis of voluntary movement exists on both sides of the body, but incomplete, and the paralysis of sensibility only on one side, and it is opposite to the side of the disease. *c.* The alteration occupying the entire thickness of a portion of a lateral half of the spinal cord,—the parts of the body situated behind it, on the same side, are paralyzed of voluntary movement, and the corresponding parts, on the other side are paralyzed of sensibility."—Pp. 30. But "as the sensitive nerve fibres, coming from one side of the body have to pass through the corresponding side of the spinal cord to go to the opposite side of the organ, it is easy to understand that an alteration occupying the entire thickness of the cord, is able to produce a paralysis of sensibility, together with a paralysis of movement in the same side of the body."—Pp. 32.

In explanation of certain pathological difficulties in connexion with crossed paralysis, our author says: "That it seems that, in some men, the sensitive and voluntary motor nerve fibres do not decussate at their usual place, and that, in consequence, some rare cases may exist in which an alteration in one side of the pons Varolii or of the medulla oblongata, will produce paralysis of the same side of the body.—Pp. 66.

*Results of experiments on the posterior columns of the spinal cord.*—In referring to these, we will endeavor to follow the order of the experiments recorded by M. Broca, as made in the presence of himself and other members of the Société de Biologie, by M. Brown-Séquard. *Section of the posterior columns* gives rise to pain and convulsive movements. Sensibility in the parts below the section *still remains*, and hence it is to be concluded, that "these columns are not indispensable to the transmission of sensory impressions;"—in fact, *hyper-æsthesia* of the lower limbs followed the section made in the lumbar region. On examining the sensibility of the divided portions of the columns themselves, it was found that while the cephalic portion was sensitive, the caudal segment was much more so—a fact which reverses all our ideas of the *direction of the nervous currents.* He found, also, that the grey matter of the cord was insensible, although, as will be seen, it is the true conductor of sensibility, which, together with power of motion, disappeared on its section in the parts below, even though the whole of the anterior columns, and about a third of the lateral columns, were left entire. In experiments again where all of the cord except the posterior columns, was divided, sensibility and power of motion disappeared in the parts below.

The hyper-æsthesia, once established in the hinder part of the body by section of the posterior columns, remains during the lifetime of the animal; and both this and the fact of sensibility of the caudal segment of these columns remaining and being excessive, are shown to be only explicable on the view that there are descending as well as ascending fibres in these columns, and that the latter are the more numerous. In fact, it would appear, from experiments made to ascertain the point, that these descending fibres run down a very considerable distance before they communicate with the grey matter which is to conduct the impressions they transmit to the sensorium. Moreover, it appears that the restiform bodies which have hitherto been regarded as transmitting impressions upwards, consist of fibres *all* of which conduct downwards. "At no epoch, perhaps, has the physiology of the nervous system been overturned by a revolution more radical or more rapid. Yesterday it was an harmonious whole, formed of undoubted propositions, ingenious explanations, and seductive theories—to-day it is a chaos. It was then a beautiful science, of which the nineteenth century was proud, and now it is a mass of ruins."—*From a Review in the London Med. Times and Gazette.*

---

A warm controversy is now going on in the London Lancet between Dr. Henry Bennett and Dr. W. Tyler Smith, regarding the existence of ulceration of the uterine neck. It commenced, by Dr. Bennett affirming that experience had entirely changed Dr. Smith's views upon the ulcerative nature of abrasions and excoriations of the cervix. In reply, Dr. Smith denies that he has changed his opinions, and states that, as regards the origin, pathological importance and the treatment of the granular state of the os uteri, he is still as much at issue with Dr. Bennett as he ever was.

The following remarks by Dr. Smith, upon the action of potassa fusa and the actual cautery, will be found interesting.

"In my experience of the results of caustic, as used by other practitioners, I have seen one or both lips of the os uteri entirely destroyed. I have seen the stump of the anterior portion of the cervix form adhesions to the anterior wall of the vagina, or the posterior lip agglutinated to the rectum, menstruation being performed with difficulty, sterility being produced, and perhaps fortunately, inasmuch as in the event of pregnancy serious lacerations must have occurred during parturition. I have known pelvic cellulitis and pelvic abscess to occur from the use of caustic potash. Cases are on record, in which perforation of the recto-vaginal cul de sac and fatal peritonitis have occurred from the same cause. I have seen cases of incipient cancer in which the sufferers had been impatient under the use of soothing measures, and had been subjected to cauterization, converted into open cancer, progressing rapidly to a fatal termination. I have known nymphomania, acute metritis, aggravated hysteria, and many weeks of pain and misery ensue upon their use. I have seen a case in which the os and cervix uteri had been destroyed by cauterization, and pregnancy occurring, the lower part of the uterus was converted into an open slough, there being no cervix to develop. The progress of pregnancy was impossible, and the patient aborted in two successive pregnancies. I have seen the os uteri destroyed in a woman dying of phthisis. At this time I have an out-patient at St. Mary's Hospital, to whom the following history belongs:

L. F—— became an out-patient of St. Mary's Hospital at the end February, 1856. She is forty-eight years of age, and has had four children. Nine years ago, she became a patient in a public institution, to which she applied on account of leucorrhœa. She remained under treatment two years, during the whole of which time strong caustics were applied at intervals. In June, 1847, the monthly periods suddenly ceased, and she has never menstruated since. Up to that time she had been regular. She subsequently became a widow. Since the stoppage occurred, she has not been free from pain in the back, and she looks in miserable health, the result, as she declares, of constant pain. There is a good deal of vaginal discharge.

On making a digital examination, the vagina was found to be a mere cæcal tube or cul de sac, not the slightest trace of the os or vaginal portion of the cervix uteri could be felt. On examining with the speculum, a cicatrix was seen at the point at which the canal of the cervix, must have closed, but it was impossible to pass the smallest probe. It appeared as if the whole of the canal of the remaining portion of the cervix had been obliterated. I happened to have at the same time under treatment a case in which sloughing of the os and cervix, followed by occlusion of the uterine canal, had occurred after an instrumental labor. The appearances presented by the two cases were very similar, except that much more of the uterus had disappeared in the case in which the infibulation and mutilation depended upon cauterization. The principle I would contend for in the treatment of uterine disease is a very simple one. It is that *no mutilation* should be practised; and cauterization by the white iron or by potassa is mutilation. These agents cannot be used in women during the child-bearing era without mutilating and destroying, more or less, portions of an organ which may be required for the purposes of menstruation, gestation, and parturition. If Dr. Bennett's method of practice has been adopted and carried out to the extent he asserts, and if the cases I have met with are seen by others in a like proportion, there must at the present time be, in this country, hundreds of women with the os and cervix uteri mutilated. I hold that in such disorders as the hypertrophy, ulceration, and induration of Dr. Bennett, the actual cautery, or caustic potash, which he tells us is the same thing, only that the use of caustic potash is unattended by the fizzing, smoke, and smell of the burning uterus, under the white-hot iron, should never pass the ostium vaginæ. Dr. Bennett speaks of assimilating the treatment of diseases of the uterus to the surgical treatment of other organs. But what should we think of that surgery which treated the disorders of the nose, eye, or ears, by agents which risked the mutilation of these organs? The uterus is the organ in which, of all other organs in the human body, mutilation should be avoided."

To these strictures, Dr. Bennett answers as follows:—

"I have no hesitation whatever in stating, in the most peremptory and decided manner, that no cases of this description, which Dr. Tyler Smith may have seen either at St. Mary's or elsewhere, were or have been cases under my care, and I defy him to prove to the contrary. I have been now many years in practice, and hundreds of my patients, poor and rich, are disseminated throughout the length and breadth of the land, but not one of them bears the trace of either mutilation or destruction of the cervix uteri. I was the first, it is true, to introduce potassa c. calce as a surgical agent in the treatment of uterine disease to the profession in this country; but I have ever taught that it ought only to be used to modify morbid vitality, not to destroy diseased or hypertrophied tissues. This, indeed, is the principle I have constantly kept in view, and on which I have constantly laid the greatest stress with reference to the whole class of caustic agents.

This rule I may mention, however, has not been adhered to by all. Dr. Simpson, of Edinburgh, with his usual surgical perspicacity, saw at once the advantage to be derived in obstinate cases from so manageable a remedy as potassa fusa, and not only adopted it, but recommended it to be used for the purpose of actually destroying part of the diseased regions of the cervix uteri, (see his collected memoirs, vol. i., page 101.) I must say that I think, and have always thought, Dr. Simpson wrong in thus departing from the rules of conduct which I had laid down; and the paper that I read at the Medical Society (see The Lancet, July, 1854) was principally written to discountenance such practice, and to bring again clearly forward my own more cautious treatment.

All this Dr. Tyler Smith knows as well as I do, and he has no right to make me responsible for the ideas and practice of others or to endeavor to damage me by implying, as he evidently does in his "Observations," that I am responsible, either personally or by precept, for the disastrous results which he says he has observed. Knowing, as he must do, that it is Dr. Simpson, and not I, who advises hypertrophy of the cervix to be "destroyed" in some cases, he ought, both in his work and recent "Observations," to have attacked, not me, but Dr. Simpson himself, or at least those of his too-enthusiastic followers who may have perpetrated the misdeeds he justly enough condemns. I presume, however, he would rather have me for an antagonist than the powerful Edinburgh professor, than whom no one is better able to defend his views and practice. *London Lancet.*

